# Responses of soil fungal community composition and function to wetland degradation in the Songnen Plain, northeastern China

**DOI:** 10.3389/fpls.2024.1441613

**Published:** 2024-09-09

**Authors:** Zhizhi Yan, Shuhan Yang, Lei Chen, Yu Zou, Yupeng Zhao, Guang Yan, He Wang, Yining Wu

**Affiliations:** ^1^ College of Wildlife and Protected Area, Northeast Forestry University, Harbin, China; ^2^ College of Pharmacy, Qiqihar Medical University, Qiqihar, China; ^3^ Halahai Provincial Nature Reserve, Qiqihar, China; ^4^ Heilongjiang Academy of Sciences Institute of Natural Resources and Ecology, Harbin, China

**Keywords:** wetland degradation, soil fungal community, composition and function, highthroughput sequencing technology, Songnen Plain

## Abstract

**Introduction:**

Wetlands are ecosystems that have a significant impact on ecological services and are essential for the environment. With the impacts of rapid population growth, wetland reclamation, urbanization, and land use change, wetlands have undergo severe degradation or loss. However, the response of soil fungal communities to wetland degradation remains unknown. It is crucial to comprehend how the diversity and population dynamics of soil fungi respond to varying levels of degradation and ecological progression in the wetlands of the Songnen Plain.

**Methods:**

In this study, high- throughput sequencing technology to analyze the variety and abundance of soil fungi in the undegraded (UD), light degraded (LD), moderate degraded (MD), and severe degraded (SD) conditions in the Halahai Nature Reserve of Songnen Plain. This study also explored how these fungi are related to the soil’s physicochemical properties in wetlands at various degradation levels.

**Results:**

The findings indicated that Basidiomycota and Ascomycota were the primary phyla in the Songnen Plain, with Ascomycota increasing and Basidiomycota decreasing as wetland degradation progressed. Significant differences were observed in soil organic carbon (SOC), total nitrogen (TN),and soil total potassium (TK) among the succession degradation stages. With the deterioration of the wetland, there was a pattern of the Shannon and Chao1 indices increasing and then decreasing. Non-metric Multidimensional Scaling (NMDS) analysis indicated that the fungal community structures of UD and LD were quite similar, whereas MD and SD exhibited more distinct differences in their fungal community compositions. Redundancy analysis (RDA) results indicated that Soil Water content (SWC) and total nitrogen (TN) were the primary environmental factors influencing the dominant fungal phylum. According to the FUNGuild prediction, Ectomycorrhizal and plant pathogens gradually declining with wetland degradation.

**Discussion:**

In general, our findings can offer theoretical support develop effective solutions for the preservation and rehabilitation of damaged wetlands.

## Introduction

Natural wetlands are crucial terrestrial ecosystems comprising just 5-8% of the Earth’s land area. Owing to their unique combination of terrestrial and aquatic features, these ecosystems boast the most diverse range of species, highest levels of productivity, and most valuable ecosystem services on the planet (Taufik et al., 2015; Zheng et al., 2013), They played an essential role in regulating the carbon cycle, maintaining global water balance, and preserving biodiversity (Liu et al., 2014). The functions of wetland areas are deteriorating in numerous regions worldwide owing to the expansion of industrialization, urbanization, and rapid population growth (Malekmohammadi and Blouchi, 2014). In the last 150 years, human activities have altered or degraded over half of the Earth’s wetlands (Sica et al., 2016), making wetland ecosystems one of the most threatened ecosystems in the world (Junk et al., 2012), and the situation of wetland degradation has become increasingly critical. In recent years, many studies have investigated the effects of wetland degradation on the diversity and abundance of soil microbes, thereby weakening the wetland functions (Wu Y. et al., 2021; Hofer, 2022; Cao et al., 2022). Wetland degradation is associated with a decrease in soil nutrients and dominant bacterial and fungal phylum. The survival of fungi is contingent upon the availability of soil nutrients, and the degradation of wetlands can result in a substantial decline in soil nutrient levels, including total carbon, total nitrogen, organic carbon, and total phosphorus, thereby impeding the growth of soil fungi (Lin et al., 2023; Cao et al., 2022),. Furthermore, soil moisture is a critical determinant of microbial community structure and enzyme activity. Wetland degradation alters soil water content, consequently inducing changes in the structure and function of microbial communities (Ma Y. et al., 2018; Wu et al., 2016; Zhao et al., 2023). Previous studies have shown that soil microbes may play a key role in determining the direction of wetland degradation (Cho et al., 2017). For instance, nitrogen-fixing bacteria and arbuscular mycorrhizal fungi contribute to the recovery of damaged wetlands by increasing the availability of nutrients in the soil and supporting plant growth (Che et al., 2019). Hence, studying the impact of wetland degradation on soil microorganisms can improve our understanding and help to predict and manage degraded wetlands.

Microbes are vital elements in wetland environments, contributing significantly to ecological functions, such as breaking down organic matter, enhancing soil quality, and recycling nutrients and carbon and nitrogen compounds (Schloter et al., 2003; Yao et al., 2000). Changes in the soil microbial community diversity and structure can reflect microbial adaptation mechanisms to habitat changes (Marschner, 2003; Segata et al., 2011). The degradation of wetlands can result in substantial alterations to the soil physicochemical properties as well as ecological processes. These alterations, in turn, affect the composition, structure, diversity, and functional roles of soil microbial communities (Li et al., 2022b). It has been shown that the spatial distribution pattern of soil fungal sites has significant habitat-dependent characteristics, and changes in environmental conditions affect the structure and function of soil microorganisms. Therefore, the diversity of soil fungal communities is often used to predict changes in soil nutrient conditions and environmental quality, and is an important indicator for evaluating ecosystem health and stability (Adamo et al., 2021; Rodriguez et al., 2019). Previous research has demonstrated that wetland degradation leads to a decline in the diversity of bacterial and fungal communities (Lin et al., 2021). Furthermore, the composition and species diversity of soil fungal communities in wetlands exhibit considerable variation across different stages of degradation. Notably, a significant decrease in soil water content, organic matter, total nitrogen, total phosphorus, available nitrogen, and available phosphorus has been observed (Li et al., 2023; Jiang et al., 2024). Mantel test analysis revealed that the bacterial community was predominantly influenced by factors such as belowground biomass, pH, soil organic carbon, and total nitrogen. In contrast, the fungal community was significantly affected by soil organic carbon, total nitrogen, available nitrogen, and available potassium. Overall, current research on the mechanism of wetland degradation has rarely been assessed until recently and needs to be urgently strengthened.

Songnen Plain is situated in the northern region of Northeast China, within a partially arid and humid climate zone. It is primarily composed of sediment from the Songhua and Nenjiang River, with the rivers displaying distinct swamp-like features. This creates wetland ecosystems with diverse structures and distinct functions. Influenced by the vulnerability of the wetland itself, and the intensification of anthropogenic interference, and other factors, the wetland in the Songnen Plain shows a trend of decreasing area and gradually increasing salinization, and the wetland is degraded to different degrees (Zhao et al., 2022). Currently, the degradation of wetlands in the Songnen Plain has attracted widespread attention, and the relevant departments have implemented local ecological restoration and reconstruction projects in important wetlands in the Songnen Plain (Tong and Lv, 2007), and the relevant studies of scholars are mainly focused on the protection of vegetation diversity (Liang et al., 2023; Wang et al., 2022), and the enhancement of wetland functions (Gao et al., 2024; Song et al., 2024), and ecological restoration of wetlands (Liu et al., 2024). However, relatively little research has been conducted on the structure of soil microbial communities, differences in their diversity, and the factors that influence these aspects. Here, we initially investigated the soil fungal community to reveal its distribution patterns and ecological functions. Our study provides a scientific basis for soil microbial communities under wetland degradation. We hypothesized that (1) soil fungal diversity would decrease with wetland degradation, (2) wetland degradation would change the structure and function of soil fungal communities, and (3) certain environmental factors would affect the soil fungal community distribution pattern. The main purpose of this study was to determine the structural and functional aspects of soil fungi under wetland degradation, offering crucial insights into the mechanisms driving this ecological process.

## Materials and methods

### Study area and sample collection

The research was conducted in July 2022 at Halahai Nature Reserve in the western region of the Songnen Plain, located in Heilongjiang Province, China (47°32’N, 123°30’E). The sample site is situated in an area with a temperate continental monsoon climate, defined by annual precipitation levels between 390 and 480 mm and an average temperature that hovers around 4° throughout the year. Soil types are characterized by marshy, marshy meadow saline, soda saline and black alkaline soils, and the dominant plants are *Carex schmidtii*, *Leymus chinensis*, *Deyeuxia angustifolia*, *Puccinellia tenuiflora*, *Carex lasiocarpa* and *Phragmites* communis, etc.

To analyze the impact of wetland degradation on soil fungal communities, we consulted historical data with local staff and investigated the vegetation type and cover of the protected area. Based on the indicators of wetland plant species composition, aboveground biomass and cover, and with reference to the wetland vegetation degradation classification (Ma W. et al., 2018) and a paper published in Ecological indicators on the classification of degradation levels in the Songnen Plain (Wan et al., 2024), we classified into four degraded areas: undegraded (UD), lightly degraded (LD), moderately degraded (MD), and severely degraded (SD). We also collected data on the vegetation types in the study (**Table 1**). Different degraded wetland soil samples were collected in July 2022. At each of the four sites, three 1 m × 1 m sample plots were established. Soil was then collected within each sample plot using a 5 cm diameter soil extractor and the five-point method, at a depth of 0-10 cm, and a total of 12 (4×3 replications) soil samples, one soil totaling no less than 200 g, were stored in an ice box and brought back to the laboratory for rapid storage in a -80°C refrigerator. One portion of the soil was used for high-throughput sequencing of fungi, and the other portion was air-dried for the analysis of soil physicochemical properties.

**Table 1:** Information of the study site.

Site	Species Composition	Above-ground Biomass (g m^-2^)	Coverage (%)
UD	*Carex schmidtii, Carex lasiocarpa, Phragmites australis, C.appendiculata*, *C.kirganica*	216.58 ± 9.95a	83.33 ± 11.55a
LD	*Deyeuxia angustifolia, Calamagrostis epigeios, Potentilla anserina, Polygonum lapathifolium,Salix linearistipularis*	291.03 ± 65.53a	75 ± 8.66a
MD	*Puccinellia tenuiflora, Potentilla anserina, Artemisia mongolica, Equisetum arvense*	329.06 ± 50.76a	81.67 ± 10.41a
SD	Extreme salinization with no vegetation cover on the surface	0b	0b

### Soil physicochemical factors

Soil physicochemical properties were referred to Soil Agricultural Analysis (Bao, 2000). Various soil properties were measured using specific methods: pH was determined through the potentiometric method, soil bulk density (SBD) was assessed using the ring knife method, soil water content (SWC) was analyzed through the drying method, soil organic carbon (SOC) was measured via the external heating-potassium dichromate volumetric method, soil total potassium (TK) was determined using the flame photometer method, soil total nitrogen (TN) was assessed through the perchloric acid-sulfuric acid nitrification method, soil total phosphorus (TP) was measured using the alkali fusion-molybdenum antimony spectrophotometric method, soil available phosphorus (AP) was determined through the sodium bicarbonate-molybdenum antimony sulfate colorimetric method, soil available potassium (AK) was analyzed using the ammonium acetate extraction method, and soil ammonium nitrogen (AN) was measured via the Nessler colorimetric method.

### Illumina MiSeq sequencing and sequence analysis

The OMEGA Soil DNA Kit (D5625-01) from Omega Bio-Tek in Norcross, GA, USA was utilized to obtain genomic DNA samples. Subsequent analysis involved storing the samples at -20°C. The quantity and quality of DNAs extracted were evaluated using a NanoDrop ND-1000 spectrophotometer (Thermo Fisher Scientific, Waltham, MA, USA) and agarose gel electrophoresis. PCR amplification of the fungal ITS1 region was carried out using the forward primer ITS1F (5’-CTTGGTCATTTAGAGGAAGTAA-3’) and the reverse primer ITS2R (5’-GCTGCGTTCTTCATCGATGC-3’). To enable multiplex sequencing, unique 7-base pair barcodes specific to each sample were incorporated into the primers. The PCR mixture contained buffer (5×), Fast pfu DNA Polymerase (5U/μl), dNTPs, both Forward and Reverse primer, DNA Template, and ddH2O. Thermal cycling commenced with an initial denaturation at 98°C for 5 minutes, followed by 28 cycles consisting of denaturation at 98°C for 30 seconds, annealing at 55°C for 30 seconds, and extension at 72°C for 45 seconds, culminating in a final extension of 5 minutes at 72°C. PCR amplicons were purified using Vazyme VAHTSTM DNA Clean Beads (Vazyme, Nanjing, China) and quantified with the Quant-iT PicoGreen dsDNA Assay Kit (Invitrogen, Carlsbad, CA, USA). Subsequently, the amplicons were pooled in equal proportions and subjected to pair-end sequencing with 2×250 bp reads on the Illlumina MiSeq platform utilizing MiSeq Reagent Kit v3 at Shanghai Personal Biotechnology Co., Ltd in Shanghai, China.

Microbiome analysis was carried out utilizing the QIIME2 software (Bolyen et al., 2019) with slight modifications following the official guidelines. Initially, the demultiplexing of raw sequence data was performed using the demux plugin, and then primer trimming was conducted with the cutadapt plugin (Martin, 2011). Subsequently, sequences underwent quality filtering, denoising, merging, and removal of chimeras using the DADA2 plugin (Callahan et al., 2016). Non-singleton ASVs were aligned using mafft (Katoh et al., 2002), and a phylogenetic tree was constructed using fasttree2 (Price et al., 2009) in 2002. Taxonomic classification of ASVs was achieved using the classify-sklearn naiüve Bayes taxonomy classifier within the feature-classifier plugin (Bokulich et al., 2018) against the UNITE Release 8.0 Database. The project accession number PRJNA1100921 has been assigned to the sequences stored in NCBI’s SRA database.

### Data statistics and analysis

Following the standardization of ASV abundances, the alpha diversity was quantified utilizing the Shannon and Chao1 indices. To visually represent the spread of fungal diversity across areas of varying degradation degrees was achieved through the application of Non-metric Multidimensional Scaling (NMDS), incorporating the Bray-Curtis dissimilarity index. A redundancy analysis (RDA) was executed to investigate the correlation between soil physicochemical properties and the dominant phyla and genera of soil fungi, facilitated by the use of CANOCO 5.0. Additionally, a Pearson correlation analysis was implemented to confirm the association between soil properties and fungal diversity indices, followed by the construction of corresponding correlation heatmaps. NMDS and RDA, we facilitated through the utilization of the vegan package within the R programming environment. The FUNGuild tool was employed to extrapolate the nutritional types of soil fungi. The examination of the soil physicochemical properties was conducted using SPSS (version 27.01). To identify variations in the soil fungal composition, the Kruskal-Wallis non-parametric test was applied. Additionally, the Analysis of Variance (ANOVA) was used to assess the significance of differences in soil physicochemical properties as well as fungal diversity among the sampled group.

## Results

### Soil physicochemical properties under various types of degraded wetlands

The research results show that there were notable variances in the soil physicochemical characteristics among UD (undegraded), LD (light degraded), MD (moderately degraded), and SD (severely degraded) with statistical significance (*p* < 0.05) (**Table 2**). As wetlands deteriorate, the pH of the soil increases and becomes basic, with the soil in SD being highly basic. Soil Organic Carbon (SOC), Total Phosphorus (TP), and Total Nitrogen (TN) significant decreasing trends. Conversely, Soil Bulk Density (SBD), and total kalium (TK) tended to increase. The differences in SOC, TN, and TP between UD, LD, MD, and SD were extremely significant (*p* < 0.01), and the differences in Available Nitrogen (AN), SWC, and SBD between UD and SD were also extremely significant (*p* < 0.01). Significant variations in TK were observed among the four degraded wetland stages (*p* < 0.01). As wetland degradation becomes more severe, there is a progressive increase in the release of carbon and nitrogen from the soil, leading to a corresponding decrease in soil carbon and nitrogen reserves. The highest levels of TK and Available Phosphorus (AP) are found in SD. The trend of AN showed a decrease from UD to MD to LD to SD. The trend for Available kalium (AK) is UD > MD > SD > LD. There were notable variations in SWC, ranging from 74% to 33%, particularly between UD and LD, and MD and SD (*p* < 0.05).

**Table 2:** Soil physicochemical properties at different stages of degradation in Songnen Plain wetlands.

Soil properties	UD	LD	MD	SD
pH	8.67 ± 0.1b	9.04 ± 0.63b	9.96 ± 0.2a	10.45 ± 0.07a
SOC (g·kg^-1^)	53.81 ± 3.67a	35.59 ± 10.28b	1.49 ± 4.99d	6.25 ± 0.1c
TN (g·kg^-1^)	5160.36 ± 184.25a	3394.16 ± 840.91b	3032.69 ± 302.9b	680.33 ± 30.66c
TP (g·kg^-1^)	682.89 ± 4.35ab	700.78 ± 65.02a	615.96 ± 37.03b	362.38 ± 14.11c
TK (g·kg^-1^)	14955.08 ± 119.12d	16203.57 ± 290.88c	18198.84 ± 204.47b	21638.83 ± 111a
AN (mg·kg^-1^)	263.85 ± 55.32a	166.32 ± 44.42b	169.4 ± 48.97b	32.34 ± 28.02c
AP (mg·kg^-1^)	5.83 ± 0.18b	10.94 ± 6.07ab	9.37 ± 1.81b	15.99 ± 1.15a
AK (mg·kg^-1^)	224.12 ± 23.66a	132.46 ± 20.38b	161.19 ± 28.38b	159.6 ± 15.23b
SWC (%)	0.74 ± 0.05a	0.58 ± 0.12b	0.52 ± 0.05b	0.36 ± 0.01c
SBD (g cm^-3^)	0.92 ± 0.02c	1.06 ± 0.08bc	1.08 ± 0.11b	1.32 ± 0.08a

Different letters indicate significant difference (p<0. 05). UD, Undegradation; LD, Light degraded; MD, Moderate degraded; SD, Severe degraded; SOC, Soil organic carbon; TN, Total nitrogen; TP, Total phosphorus; TK, Total kalium; AN, Available nitrogen; AP, Available phosphorus; AK, Available kalium; SWC, Soil water content; SBD, Soil bulk density.

### Soil fungal community composition structure under various types of degraded wetlands

**Figure 1:**
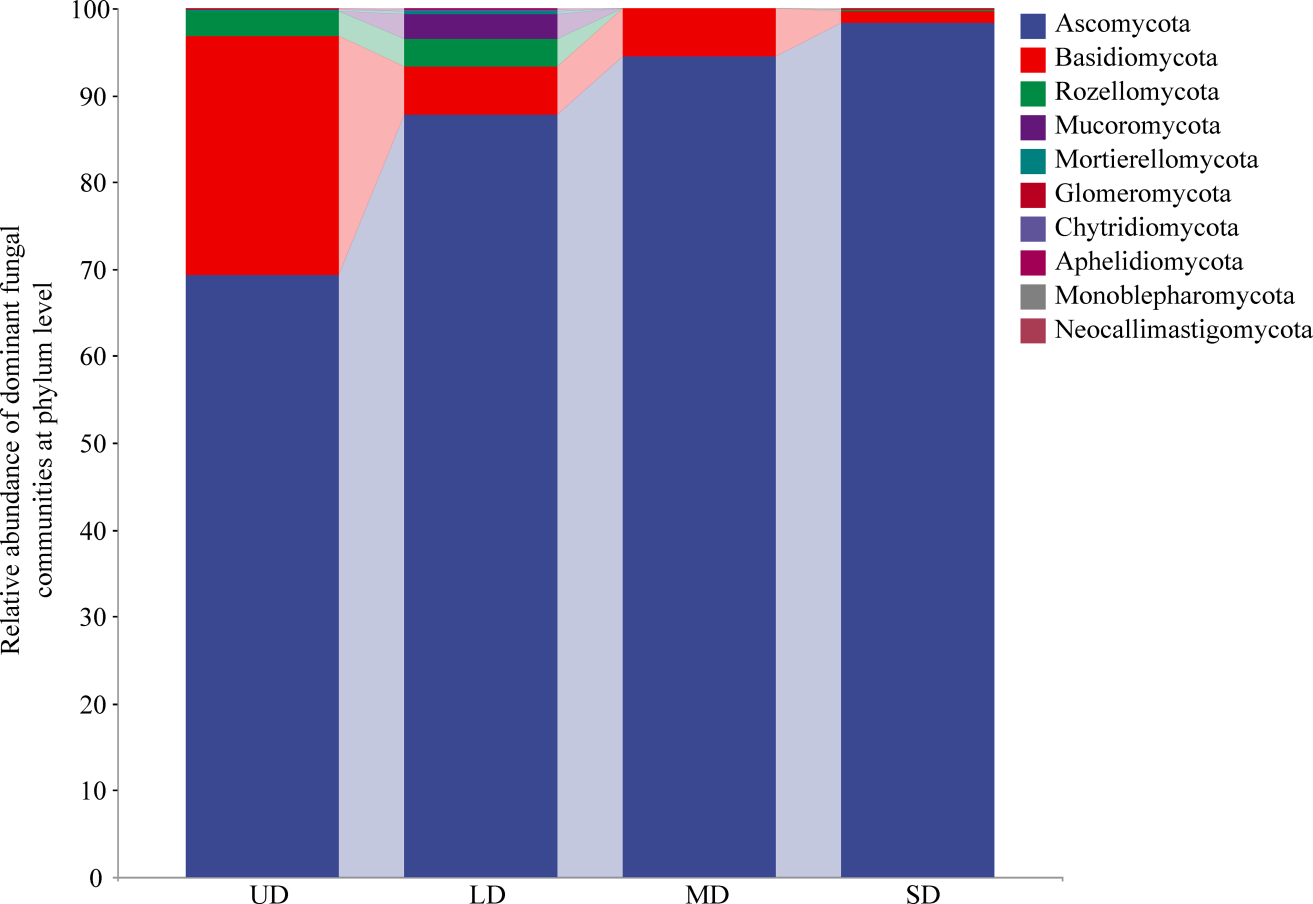
The relative abundance of soil fungal community phyla under wetland degradation. UD, Undegradation; LD, Light degraded; MD, Moderate degraded; SD, Severe degraded.

At the phylum level, 10 fungal phyla were identified in **Figure 1**. Regardless of the degradation stage, the soil of wetlands was predominantly occupied by the phylum Ascomycota, with relative abundances in the order of SD > MD > LD > UD, at 95.74%, 94.34%, 82.04%, and 65.23% respectively. In addition, the sub-dominant population in UD was *Basidiomycota* (26.27%). The relative abundance of LD greater than 1% also included *Basidiomycota*, *Cryptomycetes*, and *Mucoromycota*. The relative abundance of *Ascomycetes* (94.34%) and *Basidiomycetes* (5.56%) accounted for 99.9%, while the relative abundance of other phyla was low. The relative abundance of *Ascomycetes* and *Basidiomycetes* accounted for 97.09% in SD. Various patterns were observed in the distribution of fungal phyla between UD and SD soils, including a notable rise in *Ascomycota* (*p*<0.05) and a significant drop in *Rozellomycota* (*p* < 0.05). A Kruskal-Wallis non-parametric test was conducted on the 10 fungal groups at the phylum level, revealing notable variations in the prevalence of *Ascomycota* and *Rozellomycota* among the four degrade wetland stages (*p*<0.05) (**Supplementary Table S1**). *Ascomycota* was more abundant in UD than in MD, and even more so in SD; *Mucoromycota* was most abundant in LD compared to other sites; while Chytridiomycota was most abundant in LD compared to MD and SD.

**Figure 2:**
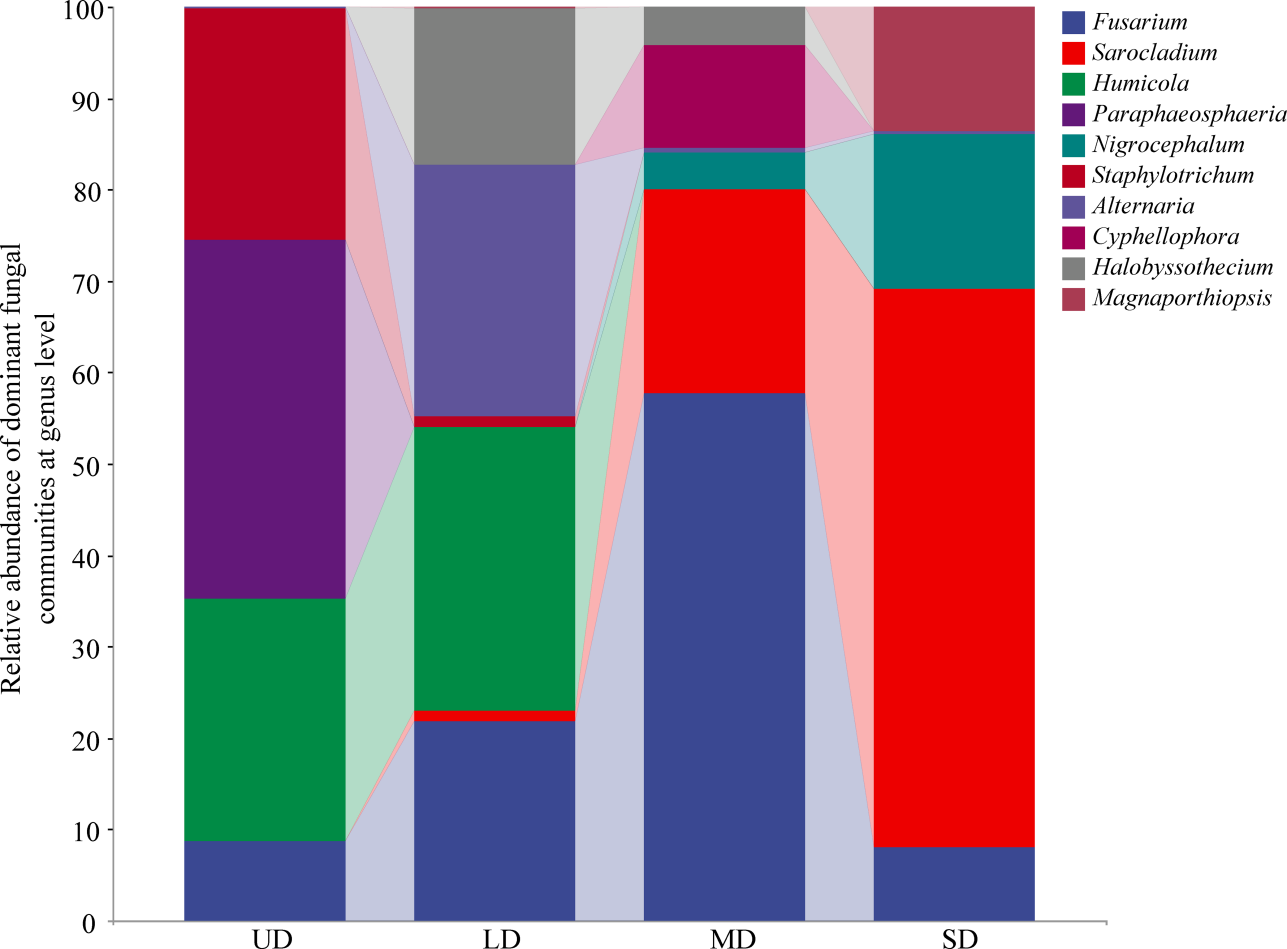
The relative abundance of soil fungal community genus under wetland degradation. UD, Undegradation; LD, Light degraded; MD, Moderate degraded; SD, Severe degraded.

At the genus classification level (**Figure 2**), *Staphylotrichum* (9.95%), *Paraphaeosphaeria* (15.34%), and *Humicola* (10.36%) are dominant in Undegraded (UD) soils; *Alternaria* (8.44%) and *Humicola* (9.60%) are dominant in Light degraded (LD) soils; *Cyphellophora* (8.17%), *Sarocladium* (15.95%), and *Fusarium* (41.47%) are dominant in Moderately-degraded (MD) soils; *Magnaporthiopsis* (6.35%), *Nigrocephalum* (7.98%), and *Sarocladium* (15.95%) are dominant in Severely-degraded (SD) soils. Different trends are also observed in the abundance of fungal genera from soils classified as UD to SD. *Fusarium* initially increases and then decreases, peaking in MD; the relative abundance of *Sarocladium* generally shows a gradually increasing trend, peaking in SD. A Kruskal-Wallis non-parametric test was performed on the 10 fungal genera at the genus level, revealing notable variations in the relative prevalence of *Sarocladium*, *Humicola*, *Paraphaeosphaeria*, and *Staphylotrichum* among the four locations (*p* < 0.05) (**Supplementary Table S2**). In the SD, the proportion of *Sarocladium* is markedly greater compared to both UD and LD soils. Conversely, the relative abundance of *Paraphaeosphaeria* and *Staphylotrichum* genera are notably higher in the UD soils when compared to other locations.

### Soil fungal community diversity under various types of degraded wetlands

**Figure 3:**
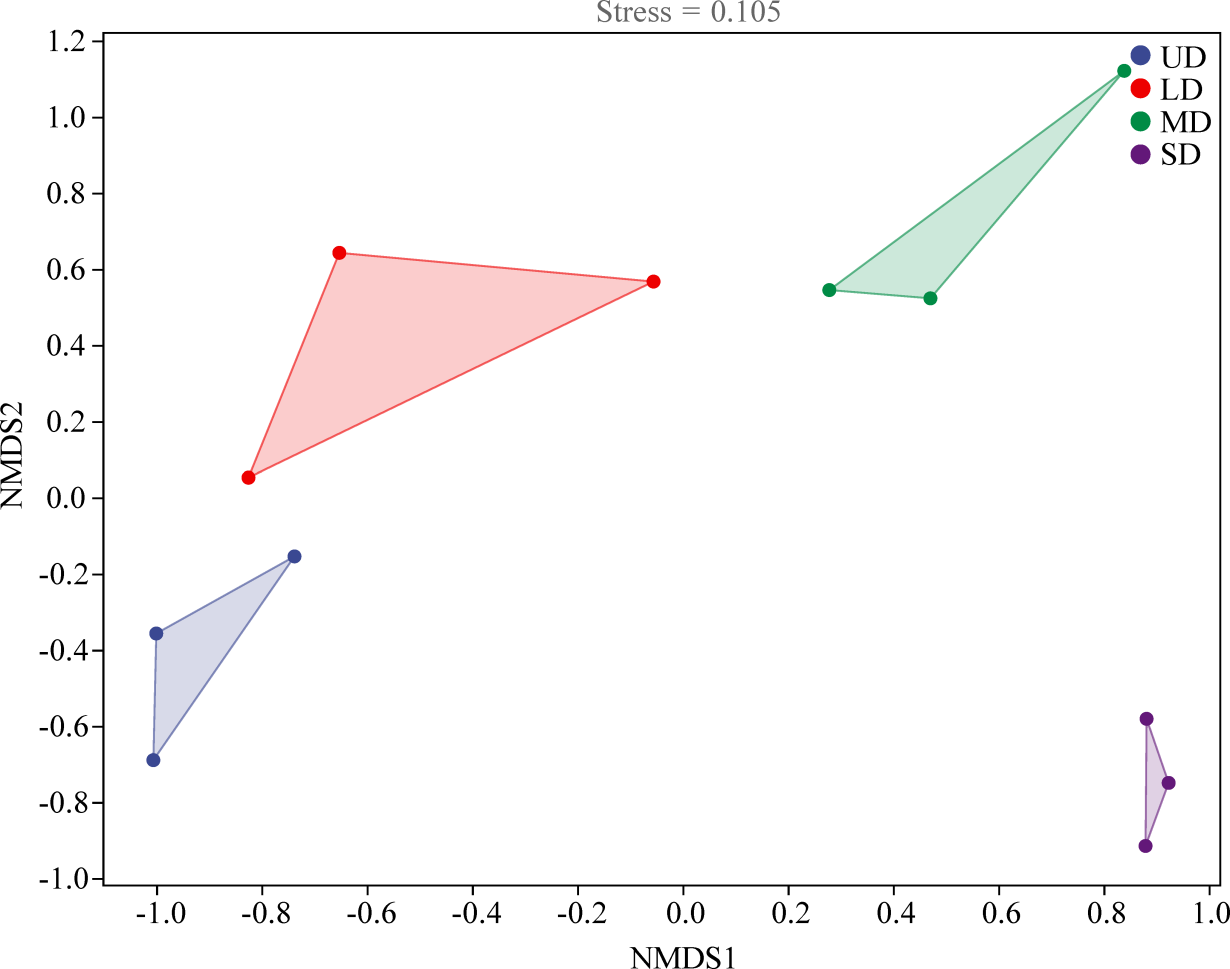
Soil fungal community alpha diversity index of different wetland degradation. UD, Undegradation; LD, Light degraded; MD, Moderate degraded; SD, Severe degraded.

**Figure 4:**
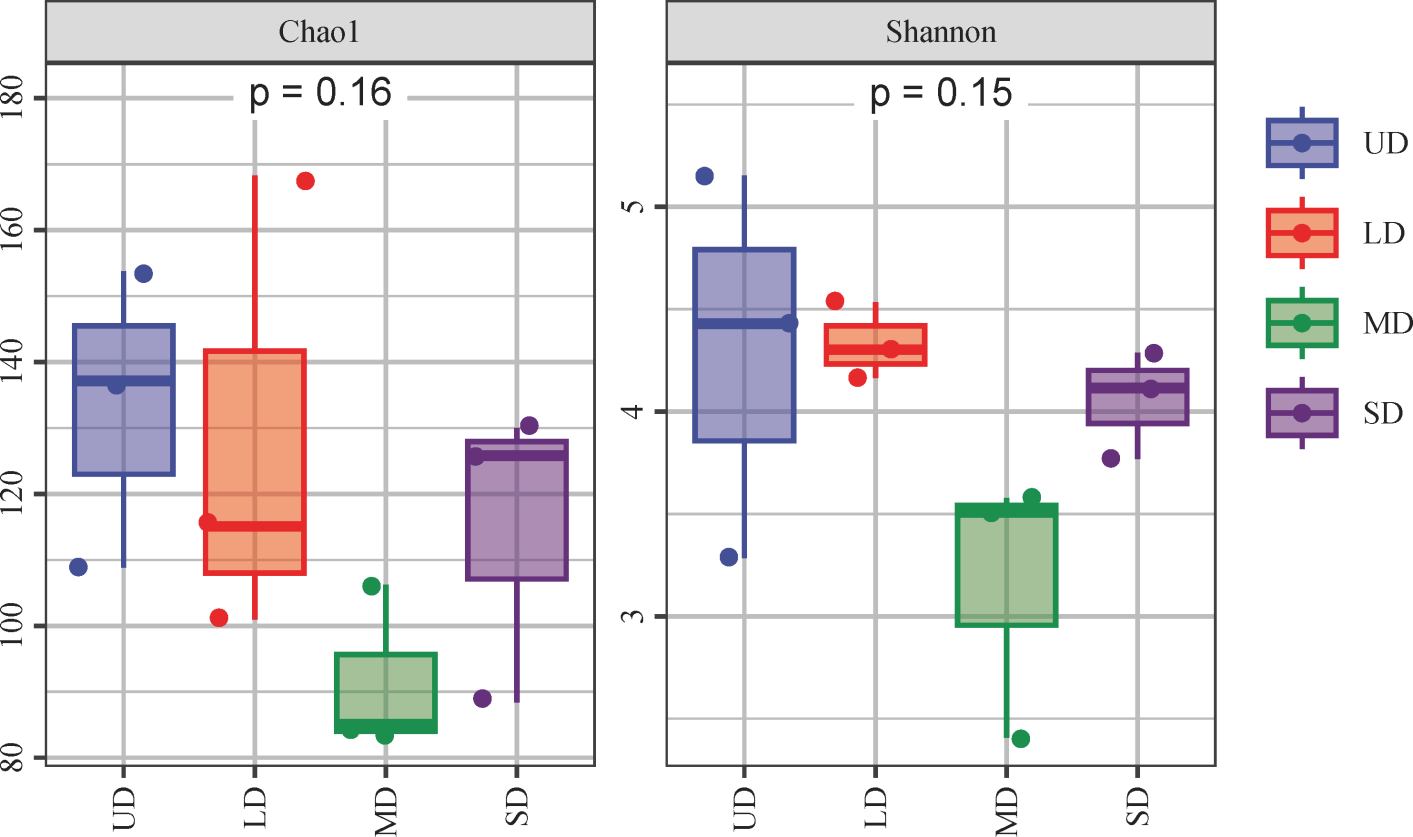
NMDS ordinations of soil fungal community structures under different wetland degradation. UD, Undegradation; LD, Light degraded; MD, Moderate degraded; SD, Severe degraded.

Comparison of the α-diversity analysis of fungal communities across four stages of wetland degradation (**Figure 3**) revealed a degree of variation in the soil fungal α-diversity indices, albeit without statistically significance (*p* > 0.05). This research aimed to assess the diversity of soil fungi across varying levels of degradation, finding that the MD stage displayed the highest values for both the Chao1 and Shannon indices, registering scores of 133.93 and 4.43 respectively. As wetland degradation increased, the Chao1 and Shannon indices initially escalated before eventually declining. The one-way ANOVA analysis of the alpha-diversity indices of soil fungi revealed a significantly higher Shannon index in both the UD and LD soils in comparison to the MD soils (*p* < 0.05). NMDS analysis was employed to examine the variability among fungal populations across areas subjected to varying degrees of degradation (**Figure 4**). The findings elucidated that the composition of fungal communities exhibited a higher degree of similarity in the UD and LD soils. Conversely, a notable increase in the fungal community composition was observed in the MD and SD regions.

### Correlation between soil physical and chemical characteristics and soil fungal communities

**Figure 5:**
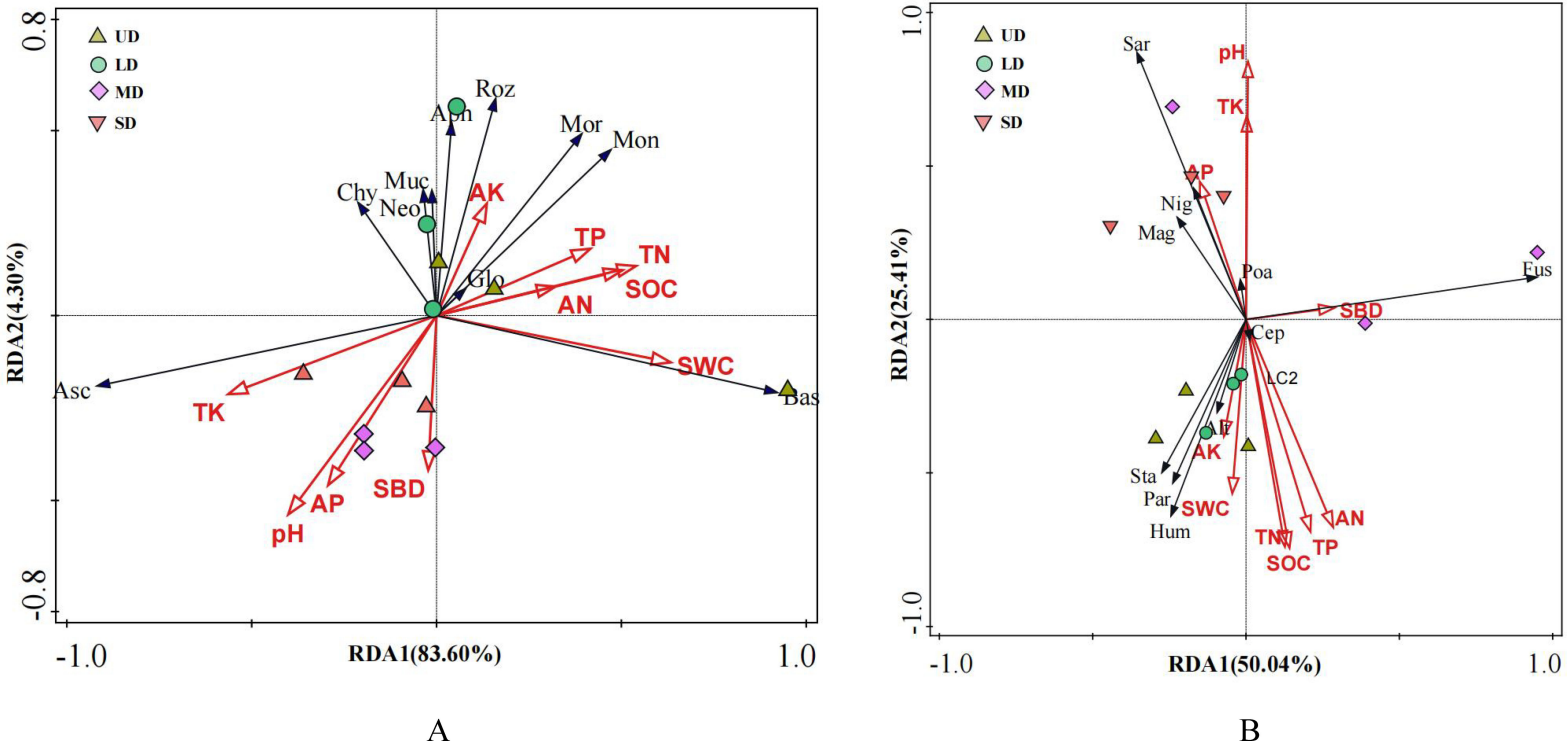
Ordination plots of the results of the redundancy analysis (RDA) based on the **(A)** fungal Phylum and **(B)** fungal Genus in relation to soil physicochemical properties. SOC, Soil organic carbon; TN, Total nitrogen; TP, Total phosphorus; TK, Total kalium; AN, Available nitrogen; AP, Available phosphorus; AK, Available kalium; SWC, Soil water content; SBD, Soil bulk density; Asc, *Ascomycota*; Bas, *Basidiomycota*; Roz, *Rozellomycota*; Muc, *Mucoromycota*; Mor, *Mortierellomycota*; Glo, *Glomeromycota*; Chy, *Chytridiomycota*; Aph, *Aphelidiomycota*; Mon, *Monoblepharomycota*; Neo, *Neocallimastigomycota*; Fus, *Fusarium*; Sar, *Sarocladium*; Hum, *Humicola*; Par, *Paraphaeosphaeria*; Nig, *Nigrocephalum*; Sta, *Staphylotrichum*; Alt, *Alternaria*; Cyp, *Cyphellophora*; Hal, *Halobyssothecium*; Mag, *Magnaporthiopsis*.

The relationship between soil fungal communities and soil environmental factors was ascertained through the use of RDA, as illustrated in **Figure 5**. The results indicated that the combined interpretations of the first and second axes in the RDA accounted for 75.13% and 67.9% of the variance, respectively. This implies a significant influence of soil physicochemical properties on the distribution and composition of fungal populations. At the phylum classification level (**Figure 5A**). The dominant fungal taxa were found to be primarily influenced by soil’s physical and chemical properties, with water content and total nitrogen being the key factors. These factors accounted for 41.5% and 28% of the variation in the dominant fungal genera, respectively. SOC, TN, TK, AN, SBD, and SWC were more correlated with Axis 1, and pH, AP, and AK were more correlated with Axis 2, with the explanation rate of the first axis reaching 66.85%. *Ascomycota* showed a positive correlation with pH and TK, while showing a negative correlation with SOC and SWC. On the other hand, *Basidiomycota* exhibited a positive correlation with SOC and SWC, but a negative correlation with soil pH. At the genus classification level (**Figure 5B**), TK, AN and pH all had notable impacts on the prevalent fungal genus taxa, with contributions of 21.8%, 19%, and 20.4% respectively, all statistically significant at a level below 0.05. TK, AP, pH, and SBD showed weaker correlation with Axis 1 compared to SOC, TN, TP, AN, AK, and SWC, which had a stronger correlation with Axis 2. The first axis in the analysis explained 48.04% of the variance. A positive correlation was observed between the fungal species Botrytis cinerea and the AK and SWC. In contrast, a negative correlation was identified in relation to soil pH, TK, and AP.

**Figure 6:**
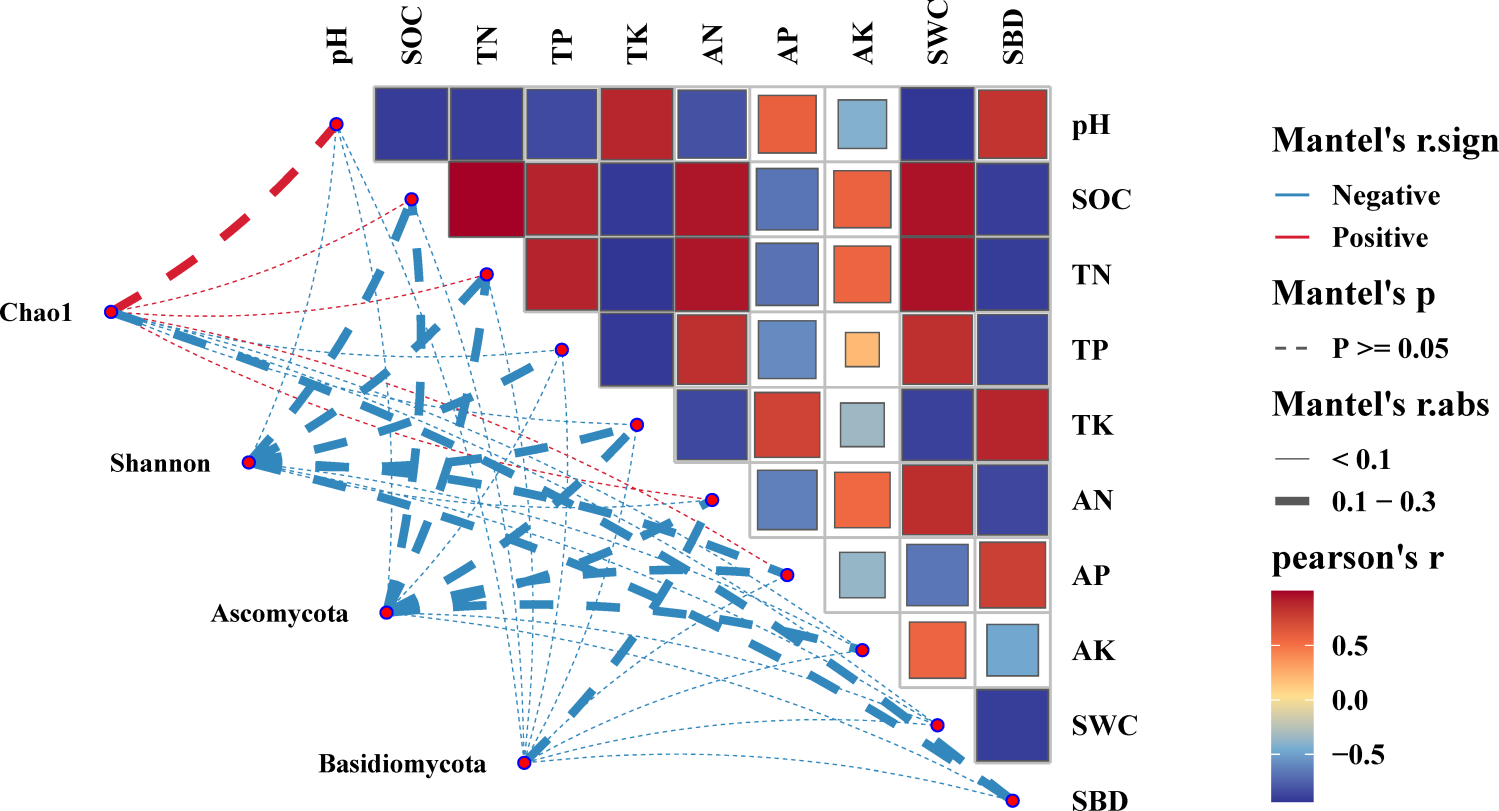
Mantel analysis of soil physicochemical properties and soil fungal communities. SOC, Soil organic carbon; TN, Total nitrogen; TP, Total phosphorus; TK, Total kalium; AN, Available nitrogen; AP, Available phosphorus; AK, Available kalium; SWC, Soil water content; SBD, Soil bulk density.

Mantel test analysis showed that the interrelation between soil physicochemical characteristics and the diversity metrics of degraded wetlands revealed a notable inverse relationship (*p*<0.05) between soil pH and the Chao1 index. Furthermore, SOC, TP, TN, AK, AN, and SWC demonstrated a positive correlation with the Chao1 index, albeit statistically insignificant (*p*>0.05). Conversely, soil pH, TP, TK, AP, and SWC manifested a negative correlation with the Shannon index, although the correlation was not statistically significant (**Figure 6**). A negative correlation was observed, however, it did not attain statistical significance (*p*>0.05). Similarly, the relationship between the presence of Ascomycota and Basidiomycota and the soil physicochemical properties attributes demonstrated a declining tendency, but it was not considered statistically significant (*p*>0.05).

### Functional prediction of soil fungal communities

**Figure 7:**
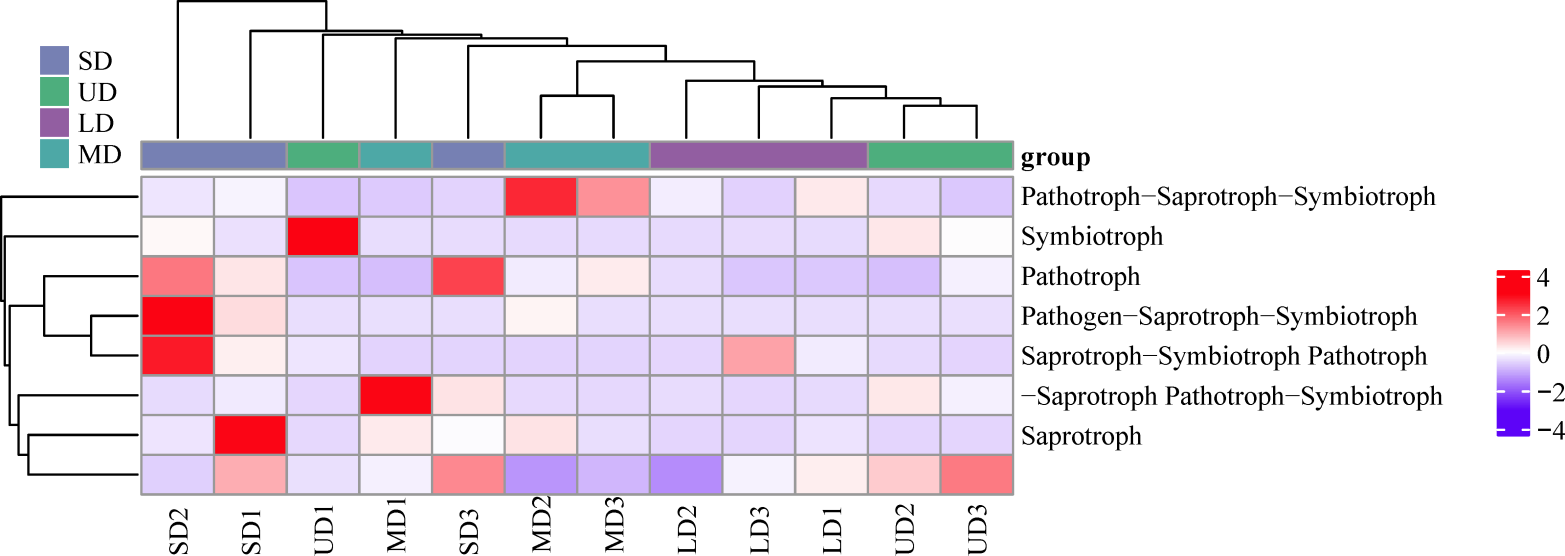
Clustered heatmap of functional prediction and annotation for primary metabolic functions of fungal communities. UD, Undegradation; LD, Light degraded; MD, Moderate degraded; SD, Severe degraded.

**Figure 8:**
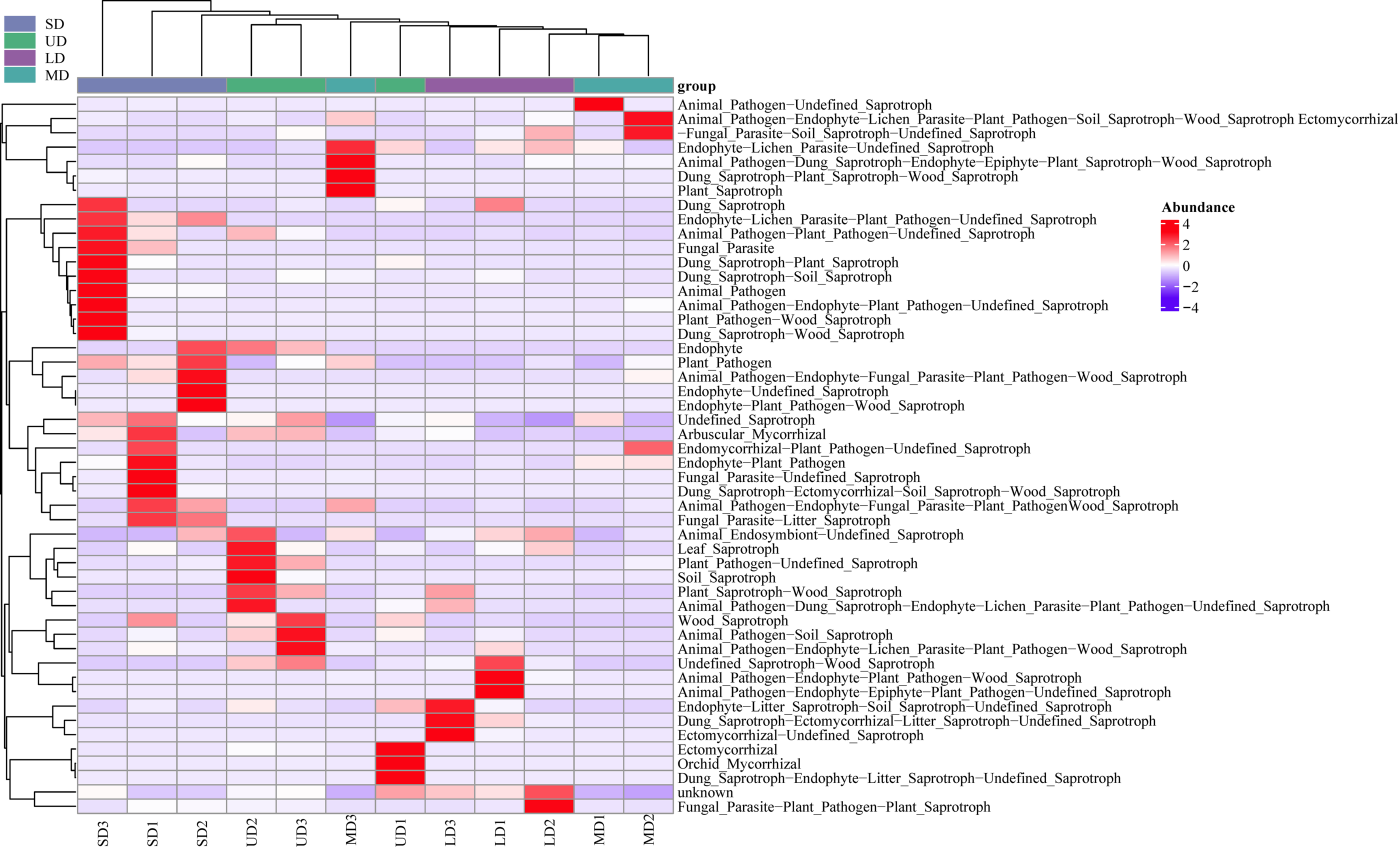
Clustered heatmap of functional prediction and annotation for secondary metabolic functions of fungal communities.

Employing the FUNGuild microecological tool to categorize and describe the usage pathways of soil fungal communities in the Songnen Plain for comparable environmental resources (**Figure 7**), soil fungi were categorized into three nutritional modes: saprotroph, pathotroph, and symbiotroph, as well as four intersecting trophic phenotypes including Pathogen-Saprotroph-Symbiotroph, Pathotroph-Saprotroph-Symbiotroph, Pathotroph-Saprotroph, Saprotroph-Symbiotroph and Pathotroph-Symbiotroph,. The dominant types were Pathotroph-Saprotroph-Symbiotroph and saprotroph, making up 35.83% and 49.06% of the total fungal community, respectively. The saprotroph was the dominant functional group in the wetland soils of the Songnen Plain, with a relative abundance of 35.60%, while the pathotroph and symbiotroph had lower relative abundances of 3.87% and 1.71%, respectively. The outcomes from a one-way ANOVA analysis revealed notable variations in the prevalence of pathotroph across different locations. Specifically, the prevalence of pathotroph in UD, LD, MD, and SD was found to be 0.30%, 0.26%, 0.65%, and 2.65%, respectively. It is worth highlighting that the pathotroph species were found to be significantly more prevalent in the SD compared to other locations. Additionally, the prevalence of Pathogen-Saprotroph-Symbiotroph was notably higher than that of MD, with UD showing significantly higher levels compared to LD and MD. There was a pattern of decline followed by growth in saprotrophic fungi. The prediction of 12 samples from 4 sample plots yielded 55 guilds, and a total of 33 species had abundances greater than 1000 in functional guilds, including Animal Pathogen-Endophyte-Lichen parasite-plant pathogen-Soil and Undefined saprotroph. The primary categories were Undefined saprotroph and Animal Pathogen-Endophyte-Lichen Parasite-Plant Pathogen-Soil Saprotroph-Wood Saprotroph, making up 34.32% and 21.76% of the total, while Undefined Saprotroph-Wood Saprotroph had a relative abundance of 7.71%, with all other functional guilds having a relative abundance of less than 5% (**Figure 8**).

Mantel test explained the relationships between fungal functional guilds and soil factors, respectively (**Supplementary Figure S1**). Ectomycorrhizal also shows a significant positive correlation with SWC (p <0.05). Leaf saprotroph is found to be significantly positively correlated with pH (p <0.05). Plant pathogen exhibits a significant positive correlation with pH, SOC, TN, TP, TK, AN, and AP (p <0.05). Lastly, Undefined saprotroph wood saprotroph is significantly positively correlated with pH (p <0.05). The abundance of these correlations is noteworthy. The prevalence of plant pathogens initially declines before escalating in correlation with wetland degradation. Concurrently, the presence of Ectomycorrhizal in wetlands is experiencing a gradual reduction, with no instances of Ectomycorrhizal being detected in SD.

## Discussions

### Effects of wetland degradation to soil physicochemical properties

Soil microbial diversity is influenced by plant diversity, pH, temperature, moisture, aeration, and land use practices (Wang X. et al., 2024; Wang S. et al., 2024). Soil characteristics are pivotal in determining the makeup of soil microbial communities (Naz et al., 2022). As degradation intensifies, plant coverage diminishes, productivity declines, and plant respiration undergoes alterations. The breakdown of apoplastic substances, along with the shallow distribution of plant roots, enhanced surface exposure, and the interplay of wind and water erosion, contribute to the rapid depletion of nutrients stored in the upper soil layer. Past research has indicated that certain soil physicochemical characteristics undergo notable alterations as wetlands degrade. Lin et al. conducted research on alpine swamp wetlands in the headwater region of the Yellow River and found that the levels of SOC and TN were significantly higher in areas that had not experienced degradation when compared to those that were mildly or severely degraded (*p*<0.05). Additionally, SWC exhibited a decreasing pattern with increasing degradation levels (*p*<0.05) (Lin et al., 2021). Li et al. (2021) discovered a strong negative correlation between SWC, SOC, and TN with the degradation gradient, all of which decreased as degradation intensified. The results of the present investigation reflected those of the previous studies. During the wetland degradation process, the increase in exposed surface area increases soil temperature, promoting the decomposition of organic carbon and nitrogenous materials in the soil. However, both types of corrosion decrease as soil moisture decreases due to rising temperatures. The reduction in the ecological function of degraded wetlands reduces the absolute amount of carbon and nitrogen combined with the ecosystem, which may be the main reason for the decline in soil organic carbon. In addition, our study showed that as wetland degradation worsened, there was a reduction in soil water content. A statistically significant difference was found between the UD and the other degraded stages (*p*<0.05). Soil organic carbon content decreases with increasing wetland degradation, while SD hold a highly significant difference compare to other sites (*p*<0.01). Therefore, it is evident that the level of moisture in the soil plays a crucial role in the degradation of wetlands (Wu G. et al., 2021), the water content in the soil is the primary physicochemical property that influences the composition and network of microbial communities. Furthermore, the research discovered a notable variation in the acidity levels of marshland grounds based on their degradation status, particularly noting a highly alkaline pH level exceeding 10 in the SD region. These results align with the research conducted by Li M et al. The process of wetland degradation caused an increase in the soil’s pH value (Li et al., 2022a). This phenomenon might due that wetland degradation led to the water content and plant community evapotranspiration reduced, causing an increase in salinity. Insufficient moisture on the soil surface also leads to serious salt accumulation, resulting in high salinity in degraded wetlands and accumulation of salt on the soil surface (Rebelo et al., 2018).

### Impact of wetland degradation on the structure and composition of soil fungi

*Ascomycota* was identified as the primary phylum in wetland soils during various degradation stages, with *Basidiomycota*, *Rozellomycota*, and *Mucoromycota* also present in relative abundances exceeding 1%. *Basidiomycota* was noted as the secondary population in UD. *Ascomycota* became more abundant from UD to SD, whereas *Basidiomycota* exhibited a declining trend in relative abundance. The primary genera varied at different degradation stages, with *Staphylotrichum*, *Paraphaeosphaeria*, and *Humicola* dominating in UD, *Alternaria*, and *Humicola* in LD, and *Cyphellophora*, *Sarocladium*, *Alternaria*, and *Magnaporthiopsis*, *Nigrocephalum*, and *Sarocladium* in MD. The dominant genera belonged to *Ascomycota* except *Alternaria*, which belonged to *Basidiomycota*. It has been noted that in various wetland environments such as alpine wetlands (Lin et al., 2021), wetlands of Poyang Lake (Wang et al., 2017), wetlands of Paleo-Great Lakes (Yu et al., 2020), and three distinct wetland ecosystems in the Western Cape Province of South Africa (Weels et al., 2022), *Ascomycota* and *Basidiomycota* emerged as the primary fungal categories in the soil, and their dominance was unaffected by different levels of degradation in those regions, mirroring the findings of the current research. *Ascomycota* and *Basidiomycota* are the primary fungi that break down organic matter in the soil, and are part of the mycorrhizal species found on land (Yelle et al., 2008). As the wetland deteriorated, the soil moisture decreased while aeration increased, creating conditions suitable for the growth of *Ascomycota* and *Basidiomycota* fungi. As soil water content decreased, the wetland soil environment underwent changes, leading to a transition in plant types from wet to mesic and arid vegetation (Wan et al., 2024). The plant species increased, the soil aeration improved, the apomictic species increased and decomposed rapidly, and the terrestrial types of *Ascomycota* and *Basidiomycota* proliferated (Heinemeyer et al., 2004). Our study revealed no significant variance across the four degradation stages. This phenomenon could potentially be attributed to the influence of the rhizosphere effect, wherein the diversity of carbon sources exuded by plant roots during succession may impact the fungal alpha diversity.

### Functional prediction of soil fungal communities

Functional annotation results from FUNGuild indicated that saprotrophs were the predominant functional trophic category of fungi present in the soil, with pathotrophs showing significant variation across diverse wetland ecosystems. Furthermore, there seems to be an increase in the relative abundance of pathotrophs in correlation with the escalating deterioration of wetland conditions. Moisture present in wetland soil, along with the gradual breakdown of organic material in the litter, fosters the widespread proliferation of saprotrophs. Given that wetland soils possess a greater water content and the organic matter within the litter decomposes got a slower pace, this creates an environment conducive to the proliferation of saprotroph. These fungi secrete hydrolytic and oxidative enzymes, facilitating the breakdown of organic matter (Lin et al., 2019; Sui et al., 2019). The majority of Ascomycetes fungi are saprophytic and excel in breaking down complex organic matter, contributing significantly to nutrient cycling (Beimforde et al., 2014). This is similar to the research results of Qi et al. (2023) on differences in fungal community structure and diversity in different seasons and habitats in the Lake Aibi saline-alkali wetland. In addition, Li et al. (2016) revealed that alpine grassland soils subjected to severe degradation contained significantly higher quantities of plant pathogens compared to non-degraded counterparts. The findings of this investigation propose that the elevated presence of phytopathogenic fungi in extensively degraded alpine meadow soils could potentially be attributed to the creation of degraded patches, a conclusion that aligns with the observations made in our own study. Therefore, wetland degradation can increase the risk of plant diseases and impede ecosystem restoration.

Furthermore, a significant positive correlation was observed between plant pathogenic bacteria and organic carbon, total nitrogen, total phosphorus, and total TK (*p*<0.05). This evidence substantiates the notion that both Ectomycorrhizal and plant pathogens necessitate nutrients for growth, and that Ectomycorrhizal fungi possess the capability to secrete enzymes for the degradation of nutrient-rich substrates to fulfill their nutritional requirements (Zhang et al., 2022).

### Linkages between soil factors and soil fungal communities

The microbial community composition and its diversity in wetland soils are influenced by numerous factors, including land use practices (Guo and Zhou, 2021), surface vegetation types (Weingarten et al., 2023), climatic change (Habekost et al., 2008), anthropogenic activities (Tang et al., 2012; Wei et al., 2019) and soil environmental factors (Chen et al., 2007). The research indicated a notable inverse relationship between pH and the Chao1 index (*p*<0.05), whereas the presence of *Ascomycota* and *Basidiomycota* was inversely associated with various soil chemical characteristics, although the association was not statistically significant (*p*>0.05). The RDA indicated that the dominant fungal taxa at the phylum level were predominantly affected by the soil physicochemical properties, with a focus on water content and nitrogen levels. These results align with the research conducted by Wu et al. (2021). The diversity of fungal communities is affected by moisture availability (Peralta et al., 2012). Fungi are highly responsive to variations in soil moisture levels, with high water content leading to anaerobic conditions that can alter the abundance of aerobic fungi and consequently affect the structure and diversity of soil fungal communities. Throughout the study, UD experienced constant water saturation, leading to low soil aeration and limited growth of aerobic fungi such as *Ascomycota* and *Basidiomycota*, whereas SD did not have water saturation, creating a suitable environment for aerobic fungi to thrive. Hu et al (Hu et al., 2014). demonstrated a strong correlation between alterations in soil organic carbon and shifts in microbial populations, leading to enhanced growth of beneficial soil microorganisms. Zhang et al. found that soil organic carbon content was correlated with fungal diversity (Zhang et al., 2024). In this study, UD had a higher organic carbon content, providing more nutrients to microorganisms. This promotes the growth, development, and reproduction of microorganisms, ultimately increasing their metabolic efficiency (Pausch et al., 2013). Fungal richness in UD was higher, with the Chao1 index being the lowest. These findings are consistent with those reported by Fang et al (Fang et al., 2021).

## Conclusions

In summary, the study revealed significant implications of marsh deterioration in the Songnen Plain on the composition of soil fungi communities. The alpha diversity indices of the fungal community exhibited a tendency to increase initially, followed by a subsequent decrease, in correlation with wetland degradation. Total Nitrogen (TN) and Soil Water Content (SWC) emerged as the principal environmental factors influencing the dominant fungal taxa. Saprotroph was identified as the predominant trophic category of soil fungi. Notably, the presence of pathotrophic fungi varied significantly across different sites. The diversity and functions of soil fungal communities are profoundly impacted by wetland degradation, which may, in turn, influence the functionality of wetland ecosystems. Notably, significant alterations in soil organic carbon and nitrogen elements have substantial effects on the process of wetland degradation. These modifications can affect crucial ecological processes, including nutrient cycling and carbon sequestration, which are indispensable for maintaining the health and sustainability of wetland environments. Therefore, in the restoration and management of wetlands, it is recommended to consider the replenishment of soil organic matter, nitrogen and water content.

## Data Availability

The datasets presented in this study can be found in online repositories. The names of the repository/repositories and accession number(s) can be found in the article/**Supplementary Material**.
