# Supplementary material for: Responses of soil fungal community composition and function to wetland degradation in the Songnen Plain, northeastern China
Source: Front Plant Sci. 2024 Sep 9;15:1441613. doi: 10.3389/fpls.2024.1441613 (PMC11416943; doi:10.3389/fpls.2024.1441613)
Supplement: Supplementary file 1 [file DataSheet1.docx]

| Phylum | Group |  |  |  |  |  |
| --- | --- | --- | --- | --- | --- | --- |
|  | UD-LD | UD-MD | UD-SD | LD-MD | LD-SD | MD-SD |
| *Ascomycota* | 0.223 | 0.052 | 0.043 | 0.362 | 0.313 | 0.915 |
| *Basidiomycota* | 0.161 | 0.169 | 0.107 | 0.974 | 0.791 | 0.767 |
| *Rozellomycota* | 0.881 | 0.012 | 0.107 | 0.040 | 0.791 | 0.767 |
| *Mucoromycota* | 0.002 | 0.991 | 0.997 | 0.002 | 0.002 | 0.988 |
| *Mortierellomycota* | 0.519 | 0.407 | 0.484 | 0.160 | 0.197 | 0.891 |
| *Glomeromycota* | 0.267 | 0.147 | 0.846 | 0.691 | 0.201 | 0.108 |
| *Chytridiomycota* | 0.371 | 0.570 | 0.710 | 0.162 | 0.590 | 0.357 |
| *Aphelidiomycota* | 0.173 | 0.934 | 1.000 | 0.196 | 0.173 | 0.934 |
| *Monoblepharomycota* | 0.227 | 0.300 | 0.300 | 0.042 | 0.042 | 1.000 |
| *Neocallimastigomycota* | 0.195 | 1.000 | 1.000 | 0.195 | 0.195 | 1.000 |

Table S1 Kruskal wallist test of the top 10 fungal in soils at different stages of wetland degradation at the phyla level

UD: Undegradation; LD: Light degraded; MD: Moderate degraded; SD: Severe degraded

Table S2 Kruskal wallist test of the top 10 fungal in soils at different stages of wetland degradation at the genus level

| Genus | Group |  |  |  |  |  |
| --- | --- | --- | --- | --- | --- | --- |
|  | UD-LD | UD-MD | UD-SD | LD-MD | LD-SD | MD-SD |
| *Fusarium* | 0.850 | 0.053 | 0.983 | 0.072 | 0.867 | 0.983 |
| *Sarocladium* | 0.210 | 0.030 | 0.006 | 0.362 | 0.138 | 0.569 |
| *Humicola* | 0.908 | 0.095 | 0.018 | 0.074 | 0.013 | 0.489 |
| *Paraphaeosphaeria* | 0.043 | 0.032 | 0.008 | 0.900 | 0.528 | 0.614 |
| *Nigrocephalum* | 1.000 | 0.432 | 0.053 | 0.433 | 0.053 | 0.187 |
| *Staphylotrichum* | 0.043 | 0.032 | 0.008 | 0.900 | 0.528 | 0.614 |
| *Alternaria* | 0.164 | 0.965 | 0.992 | 0.176 | 0.167 | 0.974 |
| *Cyphellophora* | 0.997 | 0.192 | 0.992 | 0.193 | 0.995 | 0.195 |
| *Halobyssothecium* | 0.241 | 0.500 | 1.000 | 0.591 | 0.241 | 0.500 |
| *Magnaporthiopsis* | 0.991 | 1.000 | 0.057 | 0.991 | 0.058 | 0.057 |

UD: Undegradation; LD: Light degraded; MD: Moderate degraded; SD: Severe degraded


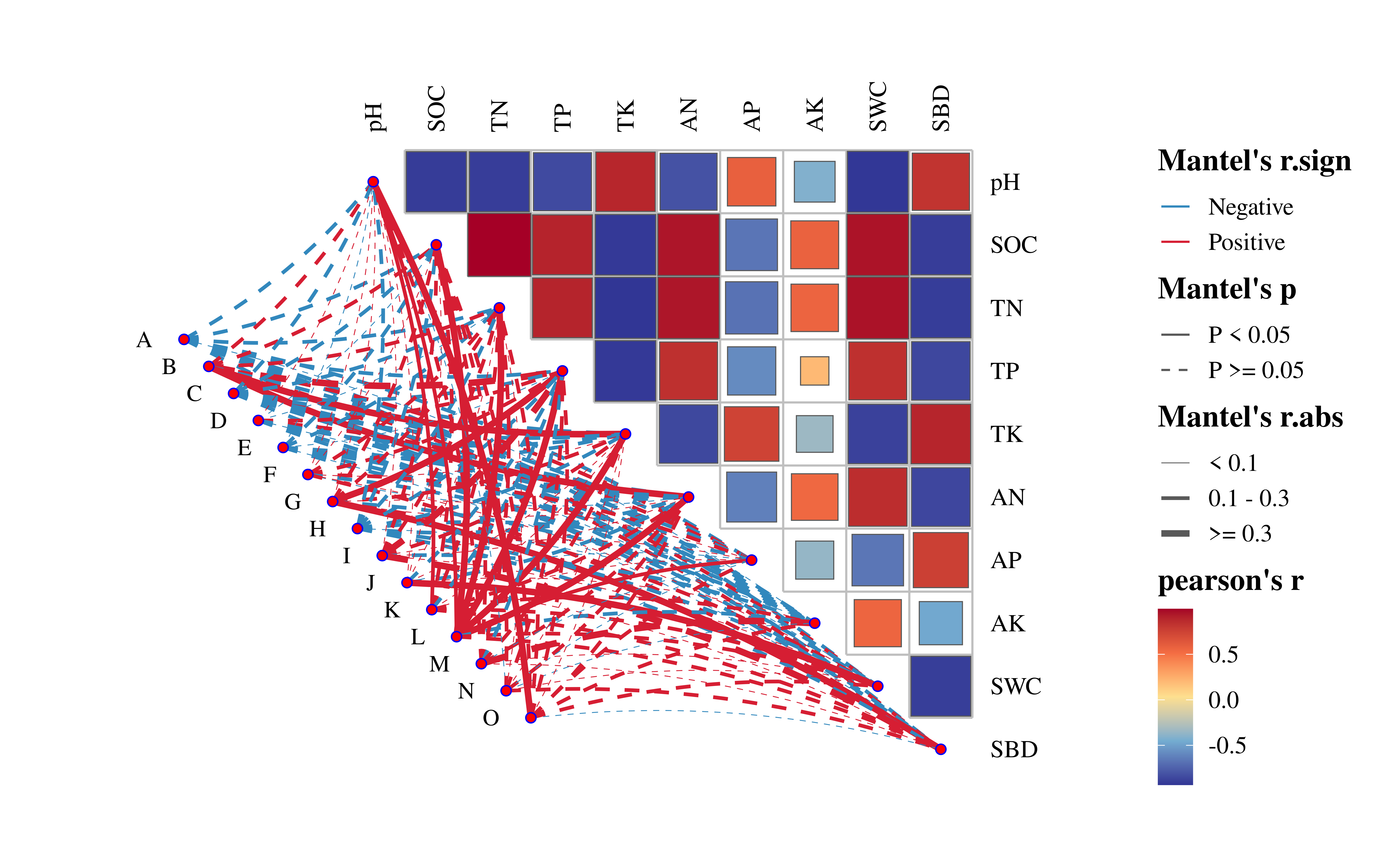


Figure S1 Correlation heatmap between soil physicochemical properties and dominant functional groups of fungi

SOC: Soil organic carbon; TN: Total nitrogen; TP: Total phosphorus; TK: Total kalium; AN: Available nitrogen; AP: Available phosphorus; AK: Available kalium; SWC: Soil water content: SBD: Soil bulk density;
